# Detection of protein-losing enteropathy (PLE) ultrasonographic imaging features in dogs using deep learning neural networks

**DOI:** 10.3389/frai.2025.1707957

**Published:** 2026-01-08

**Authors:** Anne-Kathrin Reichert, Kariem Ali, Amna Asif, Romy M. Heilmann

**Affiliations:** 1 Department for Small Animals, College of Veterinary Medicine, University of Leipzig, Leipzig, SN, Germany; 2 Department of Computer Science and Software Engineering, Lancaster University Leipzig, Leipzig, SN, Germany

**Keywords:** artificial intelligence, canine, chronic inflammatory enteropathy, deep learning, diagnosis, machine learning, model, *ResNet*

## Abstract

Artificial intelligence (AI)-based models and algorithms may aid in achieving overall more efficient and accurate diagnostics in various medical specialties. Such AI-based tools could be integrated and potentially offer advantages over currently used diagnostic and monitoring algorithms, enabling the pursue of more individualized treatment options with potentially improved patient outcomes in the future. However, very few studies exploring the potential of AI-based tools have been reported in veterinary medicine. Diagnosis and subclassification of chronic inflammatory enteropathy (CIE) and protein-losing enteropathy (PLE), requiring an integrated approach including several diagnostic modalities, remains a challenge in clinical canine gastroenterology and might benefit from AI-based tools. Thus, we aimed to use AI-based *deep learning* to develop a model that can differentiate clinical cases of protein-losing PLE from non-PLE CIE using ultrasonographic (B-mode) images. This pilot study included anonymized data extracted from the electronic medical records and diagnostic images from routine diagnostic evaluations of 59 dogs. Following several optimization steps, the final model had a high accuracy (91.57%), precision (0.9286), recall (0.9070), F1 score (0.9176), and AUC-ROC (0.9529). This model was highly sensitive and specific for the detection of ultrasonographic features associated with clinicopathologic and/or histological lesions consistent with a PLE diagnosis. Combining sonographic diagnostics with *machine learning* yielded a high degree of accuracy in PLE differentiation. The results of this study underscore the potential of integrating an AI-based model into CIE diagnostics and PLE differentiation in clinical canine gastroenterology.

## 1 Introduction

Protein-losing enteropathy (PLE) in dogs is a clinical syndrome caused by excessive protein loss through the intestines (Jablonski, 2022). This loss can result in hypoalbuminemia or panhypoproteinemia, which can lead to reduced oncotic pressure and extravascular fluid accumulation (e.g., edema or ascites) and other complications (e.g., thrombosis or thromboembolism) (Wennogle et al., 2021). PLE can be caused by several underlying diseases (Allenspach and Iennarella-Servantez, 2021; Craven and Washabau, 2019), the most common of which are chronic inflammatory enteropathy (CIE), characterized by an excessive immune response in the intestinal mucosa, primary intestinal lymphangiectasia (ILE), a dilation of the lymphatic vessels that impairs the transport of lymph and proteins, and neoplastic diseases such as intestinal lymphoma (Jablonski, 2022; Allenspach and Iennarella-Servantez, 2021; Craven and Washabau, 2019). Severe infections (e.g., endoparasites, fungal diseases) and right heart failure with intestinal lymph congestion or portal hypertension can also cause PLE and must be ruled out (Dossin and Lavoué, 2011). Non-infectious, non-neoplastic PLE can therefore be classified into primarily non-inflammatory PLE due to ILE (where impaired lymphatic drainage can lead to crypt abscesses and infiltration of inflammatory cells) and PLE secondary to CIE (where marked inflammatory infiltration impairs lymphatic drainage and increases intestinal mucosal permeability) (Jablonski, 2022; Craven and Washabau, 2019). However, both can be challenging to distinguish clinically and/or microscopically at the time of diagnosis (Jablonski, 2022; Craven and Washabau, 2019). Certain dog breeds, such as the Yorkshire terrier, Maltese, Basenji, and Chinese Shar Pei, are reported to be predisposed (Jablonski, 2022; Wennogle et al., 2021; Allenspach and Iennarella-Servantez, 2021; Craven and Washabau, 2019; Dossin and Lavoué, 2011; Equilino et al., 2015; Nakashima et al., 2015).

PLE has considerable clinical significance in canine medicine as it presents one of the potentially most critical chronic intestinal conditions in dogs, and despite intensive treatment, often has an unfavorable prognosis (Jablonski, 2022; Craven and Washabau, 2019; Dossin and Lavoué, 2011). Affected dogs often require repeated hospitalizations, medical and/or dietary treatment, and long-term management including regular veterinary visits for clinical reevaluation (Jablonski, 2022; Craven and Washabau, 2019; Dossin and Lavoué, 2011). This can represent a considerable emotional and financial burden for the owners of affected dogs. PLE is often detected at a late stage because early clinical signs can be nonspecific, and sonographic diagnosis requires experienced interpretation (Craven and Washabau, 2019; Dossin and Lavoué, 2011). The variability of the clinical findings and the lack of objective and reproducible criteria make early diagnosis particularly difficult (Jablonski, 2022; Craven and Washabau, 2019; Dossin and Lavoué, 2011). Improved diagnostic tools would, therefore, offer improved clinical decision-making, patient health and individual prognosis, and overall welfare.

Diagnosis of non-infectious, non-neoplastic PLE currently requires a stepwise approach, which integrates the results of a thorough patient history, physical examination, clinicopathologic testing, diagnostic imaging, and treatment trials to narrow down the diagnosis and exclude PLE mimics such as eunatremic-eukalemic hypoadrenocorticism, hepatic insufficiency, and protein-losing nephropathy (Allenspach and Iennarella-Servantez, 2021; Dossin and Lavoué, 2011). Documentation of structural and inflammatory intestinal lesions requires biopsies for histopathologic examination of the affected intestinal wall, preferably obtained via esophagogastroduodenoscopy in combination with ileocolonoscopy (Jablonski, 2022; Craven and Washabau, 2019; Equilino et al., 2015). These require a stable patient as a good anesthetic candidate. Management of canine PLE can be challenging and may require a multimodal approach, where dietary intervention plays a key role and other treatments must be tailored to the individual patient. However, outcomes vary, and early diagnosis and treatment initiation can improve the overall prognosis and quality of life of affected dogs (Jablonski, 2022; Nakashima et al., 2015), creating an urgent need for reliable, objective, and readily available diagnostic tools that can assist veterinarians in their assessment.

Imaging diagnostics in dogs with suspected CIE, and particularly PLE, is performed to evaluate the abdomen for possible ultrasonographic lesions consistent with CIE and/or PLE and exclude other conditions (Equilino et al., 2015; Nakashima et al., 2015; Allenspach et al., 2007; Walker et al., 2013; Wennogle et al., 2019; Leib et al., 2012). Ultrasonographic assessment may reveal changes in the intestinal wall, regional lymph nodes, and/or the presence of abdominal free fluid (Allenspach et al., 2007; Gaschen et al., 2008). The sections from the duodenum to the ileum are of particular diagnostic interest in the investigation of chronic gastrointestinal diseases causing PLE. The physiological wall diameter of the individual intestinal segments varies depending on the patient size and luminal filling status. In dogs, the physiological wall diameter of the duodenum is approximately 3–6 mm, that of the jejunum 2–5 mm, and of the ileum 2–4 mm, whereas the diameter of the colon wall is 2–3 mm (Delaney et al., 2003; Gladwin et al., 2014). PLE can cause generalized or segmental thickening of the individual wall layers (muscularis, submucosa, or mucosa) and mucosal echogenicity changes (e.g., hyperechoic striations) (Ohta et al., 2021).

**Figure 1:**
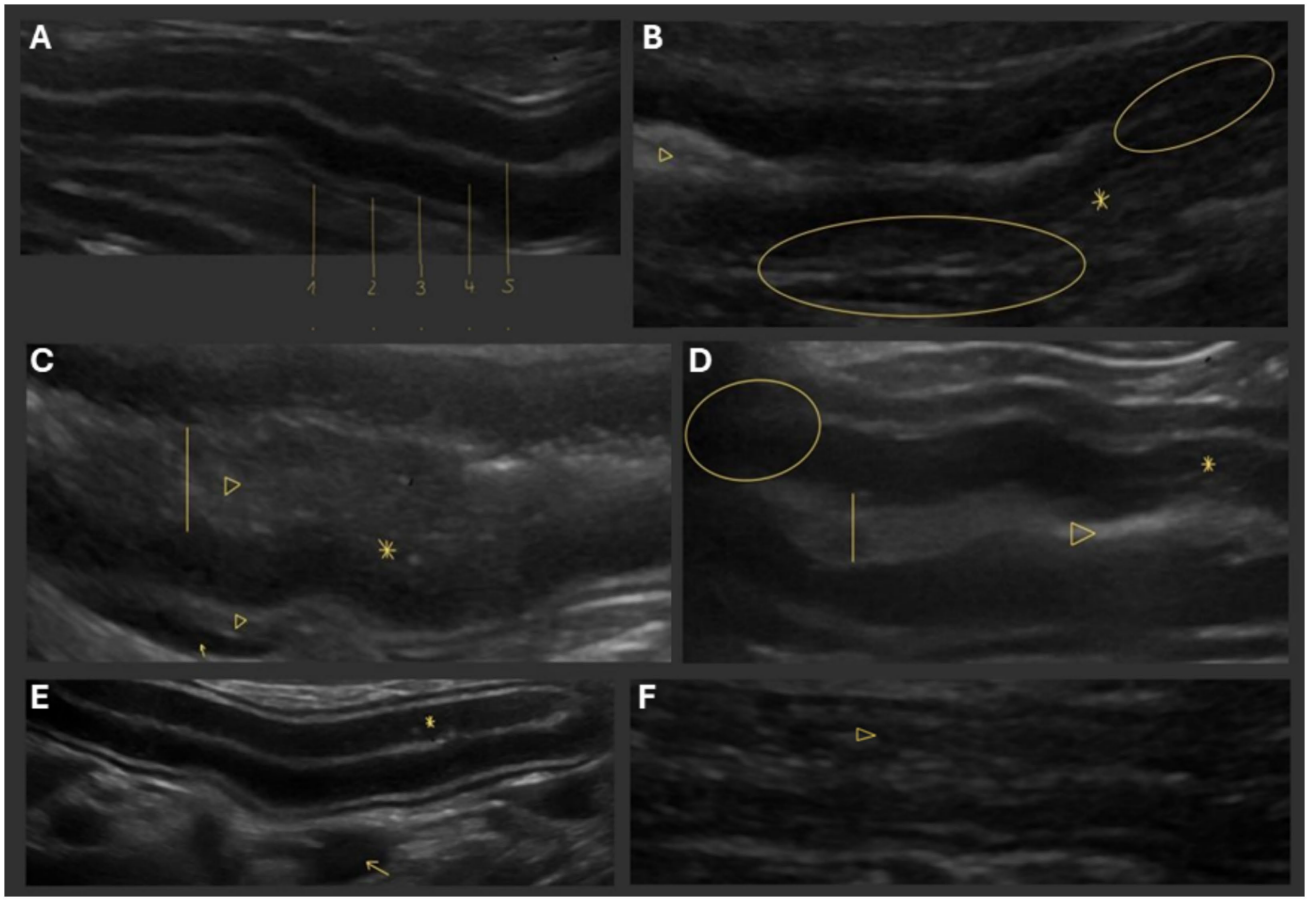
Ultrasonographic lesions in dogs with CIE and/or PLE. In contrast to **(A)** the physiological structure of the canine intestinal wall comprised of (1) serosa, (2) muscularis, (3) submucosa, and (4) mucosa, followed by (5) the intestinal lumen, **(B–F)** ultrasonographic features that can be seen in dogs with CIE/PLE but vary greatly in terms of sensitivity and specificity include intestinal wall thickening, mucosal hyperechoic speckles and striations (asterisks; **B–E**), corrugation and dilation of the intestinal lumen (arrowheads and vertical lines; **B–D,F**), disrupted intestinal wall layering (circles; **B,D**), and accumulation of free fluid in the abdomen (arrows; **C,E**).

Mucosal speckles are irregular, focal hyperechogenicities within the mucosal layer of the intestinal wall (Figure 1) that indicate an altered mucosal composition or protein and lipid deposition and can be seen with PLE, but these are a non-specific finding (Ohta et al., 2021; Nisa et al., 2019). Mucosal striations are radial hyperechoic lines within the intestinal mucosa, which are caused by lymph and fat deposits resulting in fibrotic remodeling and are a characteristic sign of impaired lymph drainage in the context of PLE (Ohta et al., 2021). Lymphadenomegaly, disruption of the physiological layering of the intestinal wall, and accumulation of free fluid in the abdomen due to hypoalbuminemia can also indicate pathology but are not specific for CIE or PLE (Equilino et al., 2015; Salavati Schmitz et al., 2019). Ultrasonographic findings vary in CIE and PLE patients and must always be interpreted in the context of clinical and laboratory diagnostic parameters (Gaschen et al., 2008; Salavati Schmitz et al., 2019).

Digital health applications and artificial intelligence (AI) tools have the potential to significantly aid in improving the standard of care in both human and veterinary medicine, particularly in diagnostic algorithms, individualized treatment options, and therapeutic monitoring (Nguyen et al., 2022; Stafford et al., 2022; Chen et al., 2025). The tools, if adequately trained and passing rigorous validation, may enable an enhanced analysis of complex data sets (e.g., within a very short time) and integrated detection of features in standardized imaging diagnostics (Reagan et al., 2022; Kathrani et al., 2024).

Standardized diagnostic and management algorithms involving AI-supported models have not been evaluated or established in canine PLE but might bear clinical potential given the complex etiology, varied clinical presentations, and required stepwise integrative diagnostic and treatment plan. The present study addresses this gap by developing, validating, and testing an AI-based model that assists in analyzing sonographic features to facilitate the differentiation between PLE and non-PLE CIE. We hypothesized that an AI-assisted model provides a reliable tool that can enable accelerated and precise (sensitive and specific) imaging detection of PLE lesions in dogs with suspected CIE, and following a training and validation phase, can aid in distinguishing PLE and non-PLE CIE cases. Thus, the study aimed to develop a model that uses AI-supported deep learning to differentiate PLE based on ultrasonographic images of the intestinal wall and peri-intestinal tissue in CIE and/or PLE suspected dogs.

## 2 Materials and methods

### 2.1 Ethics

Anonymized data from archived electronic medical records (EMR) of clinical patients presented between March 2019 and January 2021 for routine diagnostic evaluation to the Small Animal Internal Medicine Service of the Department for Small Animals were considered for inclusion in this retrospective case–control study. At the time of clinical admission and diagnostic evaluation of the patient, pet owners give their written consent on the admission form of the Small Animal Clinic of the Leipzig University College of Veterinary Medicine to the use of anonymized data, images, and surplus biological specimens of their dog for research and teaching purposes. This form has been reviewed and approved by the Ethics Committee of the Leipzig University College of Veterinary Medicine.

### 2.2 Patient data

Retrospective anonymized EMR data from a total of 157 dogs with chronic inflammatory enteropathy (CIE) were evaluated, and these dogs were categorized as either PLE or non-PLE CIE cases. This group assignment was derived from the dog’s medical and dietary history, signalment (breed, sex, neuter status, body weight, and age), physical examination findings, laboratory diagnostics (hematology, blood biochemistry panel with electrolytes, urinalysis, fecal examination, and B vitamins, pancreatic markers, and resting cortisol measurement), diagnostic imaging results, sequential diagnostic treatment trials and, in some dogs, gastrointestinal endoscopy with biopsies for histopathological examination. If indicated, additional diagnostics (e.g., ACTH or bile acid stimulation test, urine protein-to-creatinine ratio, tissue molecular diagnostics) were performed at the discretion of the attending clinician.

Sonographic B-mode still images of relevant small intestinal sections (obtained as part of the routine diagnostic evaluation of the dogs) were selected and cropped to predominantly depict the relevant structures. Smaller portions of adjacent organs (e.g., kidneys, mesenteric lymph nodes, liver, or spleen) were left in the images during the initial training phase. Measurement markers or annotations within the images were removed to prevent interference with the recognition of specific features during the training and/or validation phase. Dogs for which adequate ultrasonographic images of the gastrointestinal tract were not available for analysis were excluded from the study.

### 2.3 Image selection, pre-processing, and heatmap generation

The dataset was split into training, validation, and testing sets, comprising 70%, 10%, and 20% of the data, respectively. This split prior to data augmentation served to avoid augmented images being spread across all three different dataset splits and possibly inflating or yielding inaccurate performance estimates. Patient-level separation–although a good practice–was not performed due to the small size of the dataset. Instead, it was ensured that only non-augmented images were found across the splits. Potential inflation or inaccurate performance estimates were avoided as multiple images from the same dogs were separate crops of different regions of the small intestinal wall and did not closely resemble one another.

Data augmentation was performed for training the model due to varying numbers of adequate, high-quality B-mode images available to assess sonographic features in affected dogs (Gaschen et al., 2008; Salavati Schmitz et al., 2019). The pre-processed anonymized images were assigned to one of two separate datasets based on the final classification of the dogs as either PLE or non-PLE CIE. A machine learning (ML) model was then trained to distinguish these two patient groups with high diagnostic accuracy.

To increase the diversity and amount of the training data and ensure robust learning of the ML model to detect general features rather than noise, a specific set of affine transformations was applied to the original images. This process also served to address class imbalance by generating additional examples for the minority class (PLE) until it matched the size of the non-PLE class. An average of 3–8 augmented versions were generated for each original image, applying the following transformations randomly to introduce variation while preserving fundamental patterns: (1) rotation: images were randomly rotated within a full range of 0–360°; (2) zooming: a random zoom factor was applied ranging from 0.8 (zoom-out) to 1.5 (zoom-in); and (3) brightness: the pixel intensity was randomly adjusted with a factor ranging from 0.5 (darker) to 1.5 (brighter). Only the training dataset was augmented, while the validation and testing datasets contained only the original, un-augmented images to avoid introducing performance inflation and inaccurate results.

Images initially containing additional structures, such as the spleen or kidneys, were later removed due to presenting a source of interference for training the model. To further improve the accuracy of the model, additional processing was performed by subjecting each anonymized image to up to four iterations of cropping and pre-processing, followed by retraining of the model. These steps yielded an original PLE dataset with 200 images and a non-PLE CIE dataset including 387 images. Following an initial validation, the image data was further optimized to improve the performance of the model (e.g., targeted cropping to the intestinal wall, exclusion of insufficiently visible intestinal wall layers). This optimization resulted in an expansion of the PLE dataset from 200 to 214 and finally 227 images, and a reduction of the non-PLE CIE dataset from 387 to 190 and finally 194 images.

A preprocessing pipeline was set up using PyTorch, in which all images before entering the model were resized to 224 × 224 pixels to match the input size expected for model architectures such as *ResNet* and *EfficientNet-B0*. The images were then normalized using the mean and standard deviation of the ImageNet dataset, due to using a pre-trained *ResNet* model that is trained on ImageNet.

A subset of sonographic images and associated *heatmaps*—generated through the ML model—were manually evaluated in a blinded fashion (A-KR and RH) and areas with features consistent with PLE were manually marked based on expert consensus. These manual markings were compared with the heatmaps of the final models to evaluate each model’s ability to correctly identify affected regions and/or relevant lesions.

### 2.4 Model selection, training, and validation

Models were selected based on recent scientific literature (Kathrani et al., 2024; Saber et al., 2025; Yuan et al., 2025), in which *deep learning* was applied to sonographic images (training) and were then tested (validation). Four pre-trained *convolutional neural networks* (CNNs), *MobileNetV2*, *AlexNet, EfficientNet-B0*, and *ResNet50*, were utilized and evaluated for their performance in classifying sonographic images to distinguish PLE from non-PLE CIE. For each model, accuracy, precision, and recall were evaluated, as well as the F1 score and the *area under the curve* determined using the *receiver operating characteristic* (AUC-ROC). A high F1 score reflects a model that makes few false-positive and few false-negative predictions, and the AUC-ROC indicates how well the model distinguishes between positives and negatives (model selectivity) (Yuan et al., 2025; Ali, 2025).

### 2.5 Hyperparameter tuning and model adjustment

The selected AI model was modified to optimize the classification performance (differentiation between dogs with PLE vs. non-PLE CIE) and improve the accuracy and transferability of the model to new, unknown clinical cases (i.e., dogs with suspected CIE). These steps focused on fine-tuning important parameters in the AI learning process (hyperparameter tuning). Selecting the correct learning rate was the key factor, as models learn inaccurately when the learning rate is too high (overfitting), whereas training is inefficient if the learning rate is too low. Thus, various intermediate values were tested to select a learning rate that offers a good balance between learning speed, accuracy, and stability for AI training (Ali, 2025; Tetko and Clevert, 2025). Several techniques served to avoid overfitting. These included modification of the training images (e.g., rotation, zoom-in, and adjustment of brightness) to increase diversity of the data. In addition, mathematical methods such as L2 regularization and dropout were applied to prevent weights in the model from becoming too large and ensure the model gains robustness by randomly deactivating certain connections in the network during training (Clara et al., 2024).

**Figure 2:**
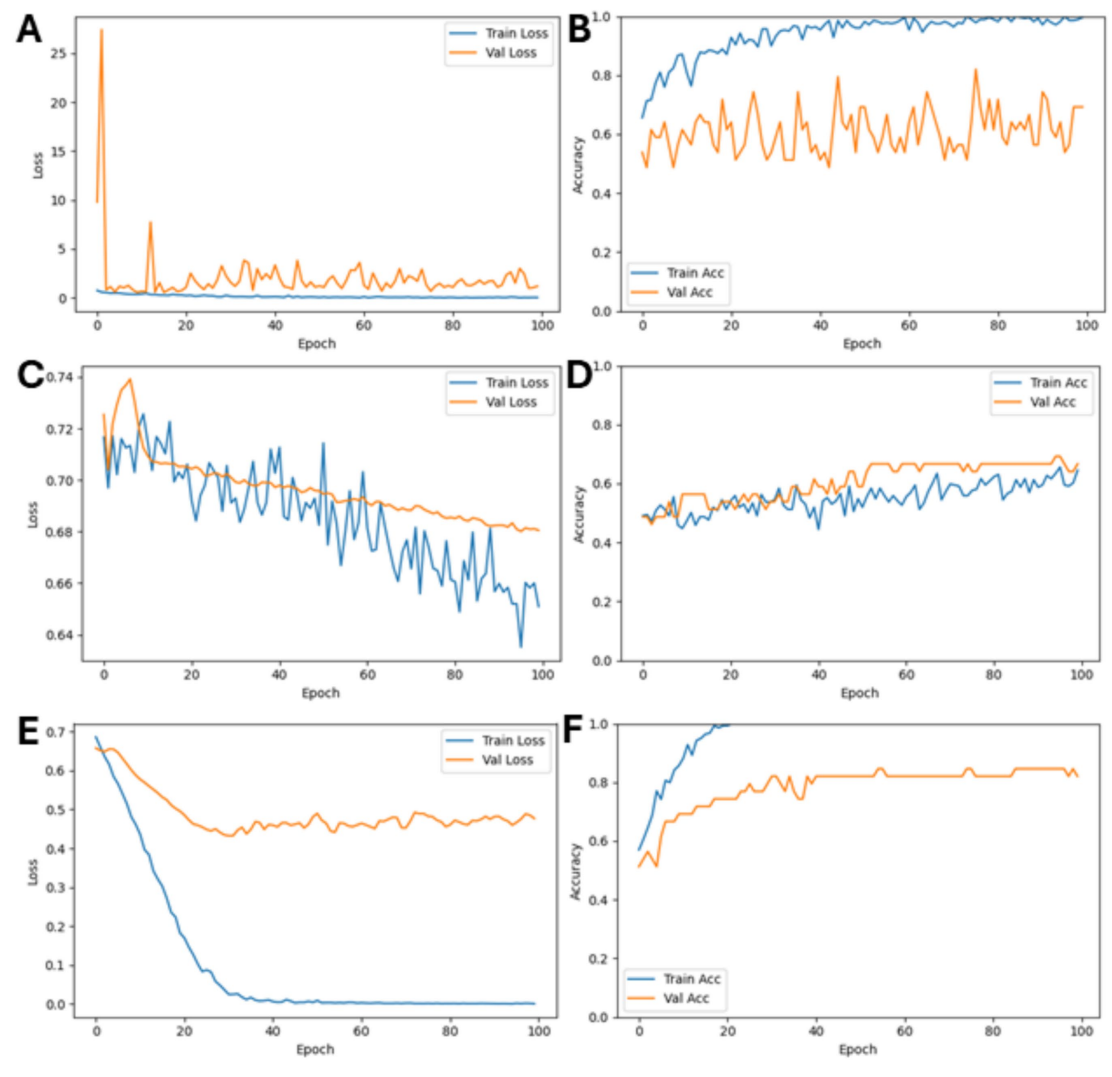
Optimization of the learning rate and success of training. While rapid overfitting and very unstable training of the selected *ResNet50* model were seen with a learning rate of 1.0 × 10^−3^ **(A,B)** and very little training with underfitting of the model at a lower learning rate of 1.0 × 10^−7^ **(C,D)**, the optimal learning rate for model training was identified as 5.0 × 10^−6^ **(E,F)**.

The final classification layer of each chosen model was adjusted from its original output dimension to a single output neuron, producing a score in the range from 0 to 1. A threshold of 0.5 was applied, classifying values above the threshold as “PLE” and below this threshold as “non-PLE.” Due to computational constraints, hyperparameter tuning was performed only on the single best-performing model identified during initial experiments (i.e., *ResNet*) with default settings (e.g., the standard Adam learning rate of 1.0 × 10^−3^ across all four architectures). To tune the learning rate of the Adam optimizer, upper and lower bounds were tested to limit the search space before determining an ideal value. An iterative process of refinement served to identify the optimal learning rate for the model training (Figure 2). To regularize the model and prevent overfitting, regularization techniques, including L2 weight decay (*λ* = 1 × 10^−3^) and a dropout layer (*p* = 0.5), were added in the classification head.

### 2.6 Annotated heat maps

Heatmaps visualize regions that are classified as particularly relevant, which can be accurately assessed by using index colors (red for high relevance and blue for low relevance). To evaluate the accuracy of the model in selecting or highlighting image areas with patterns or features consistent with PLE, the test data set (i.e., manually annotated *regions of interest*, ROIs) was compared with the heatmaps generated by the ML model (Ali, 2025). Sensitivity and specificity of the annotated images to distinguish PLE from non-PLE CIE were calculated based on dichotomous outcomes.

### 2.7 Application using a PLE detector (digital health application)

Following the training of the model, a website was designed where various sonographic images could be uploaded for testing purposes. The aim was to assess the efficiency of the final model’s classification (differentiation between PLE and non-PLE CIE) using new, unknown data and to test the potential practical applicability of the AI-guided application. Seventeen ultrasonographic still images from other sources, including 7 dogs with PLE and 10 non-PLE cases, were tested for the correct classification by using this application.

## 3 Results

### 3.1 Patient population

Considered for inclusion in the study and analysis were EMR data from 157 dogs; 42 dogs were diagnosed with PLE (PLE group) and 98 dogs with CIE lacking any clinicopathologic and/or histological evidence of PLE (non-PLE CIE group). For the remaining 17 dogs, definitive assignment to one of these two groups (PLE vs. non-PLE CIE group) was not possible based on retrospective EMR evaluation, including follow-up for some dogs, and these dogs were excluded from further analyses. Of the 140 dogs with complete EMR data, an additional 81 dogs were excluded due to (a) lack of access to (*n* = 74) or availability of adequate ultrasonographic still images (*n* = 7) of the gastrointestinal tract.

The 59 dogs that could be included in the study comprised 22 dogs in the PLE group and 37 dogs in the non-PLE CIE group (Table 1). From this cohort, a total of 421 sonographic B-mode still images were included in the final analysis (PLE group: *n* = 227, non-PLE CIE group: *n* = 194), with 1–31 still images (median: 6 still images) per dog.

**Table 1:** Characteristics of the dogs included in the study (*n* = 59).

Parameter	PLE	Non-PLE CIE	*p*-value
N	22	37	—
Patient characteristics			
Age (in years)	8.5 [3.8–11.1]	4.0 [1.8–9.4]	0.0568
Body weight (in kg)	11.1 [6.1–24.9]	13.2 [5.7–29.7]	0.5153
Sex			
Female	8 [36%]	14 [38%]	0.9098
Male	14 [64%]	23 [62%]	
Reproductive status			
Intact	16 [73%]	21 [57%]	0.2148
Neutered	6 [27%]	16 [43%]	
Breed			
Pure-bred	22 [100%]	37 [100%]	—
Mixed breed	0	0	
Serum biochemistry variables			
Albumin (in g/L)	**21 [13–25]**	**34 [32–37]**	**<0.0001**
Hypoalbuminemic	21 [95%]	0	**<0.0001**
Normoalbuminemic	1 [5%]	37 [100%]	
Total protein (in g/L)^a^	**39 [33–52]**	**65 [60–66]**	**<0.0001**
Hypoproteinemic	17 [81%]	0	**<0.0001**
Normo−/hyperproteinemic	4 [19%]	37 [100%]	

a Available from 58 dogs; values in bold font reflect statistically significant differences or associations. CIE, chronic inflammatory enteropathy; PLE, protein-losing enteropathy.

Summary statistics are presented as medians [interquartile ranges] or *n* [%].

The first set of altogether 587 images focused on intra-abdominal areas that included relevant intestinal segments and peri-intestinal tissues. An initial validation test showed an accuracy for detecting non-PLE CIE cases of 80%. The detection rate for PLE cases was lower, prompting further optimization that yielded a total of 421 images.

### 3.2 ML models for PLE diagnostics

**Figure 3:**
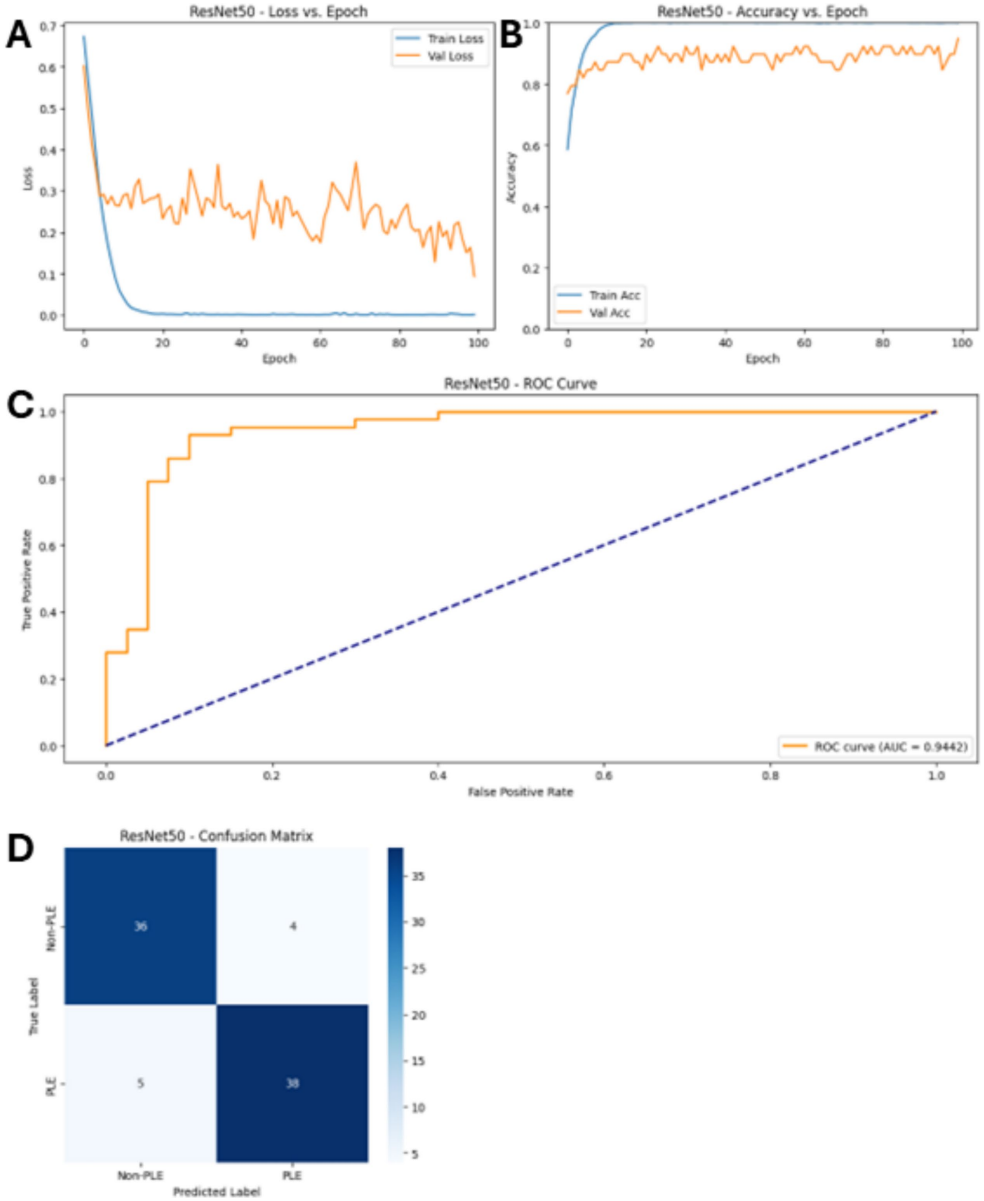
Performance of the *ResNet50* model. Shown are the results graphs for **(A)** Training and validation loss. **(B)** Training and validation accuracy. **(C)** The AUC-ROC. **(D)** The confusion matrix with the tuned hyperparameters. Note that these refer to the non-custom model.

*MobileNetV2* achieved high accuracy and precision, strong recall, and a high F1 score (Table 2). The AUC-ROC was at a high level, indicating reliable classification ability. *MobileNetV2* was the second-best model, which is considered lightweight and requires low computing power. This model outperformed larger models such as *AlexNet*. *AlexNet*, the largest model, showed the weakest performance. Accuracy, precision, and recall were moderate, resulting in the lowest F1 score and AUC-ROC. *EfficientNet-B0* delivered results with moderate accuracy, precision, and recall, and consequently, a good F1 score and AUC-ROC. *ResNet50* outperformed all other models with the highest accuracy, precision, recall, and the best F1 score and AUC-ROC (Figure 3), yielding a sensitivity of 96.7 and 79.5% specificity on a test set of 69 images (30 PLE, 39 Non-PLE CIE). This made *ResNet50* the most powerful pre-trained model for this application (Table 2).

**Table 2:** Evaluation of four different ML models.

Model	Accuracy	Precision	Recall	F1 score	AUC-ROC
*ResNet50*	89.16%	0.9048	0.8837	0.8941	0.9442
*MobileNetV2*	87.95%	0.9024	0.8605	0.8810	0.9349
*EfficientNet-B0*	85.54%	0.8780	0.8372	0.8571	0.9192
*AlexNet*	81.93%	0.8333	0.8140	0.8235	0.8994

Summarized are the accuracy, precision, recall, F1 score, and *area under the curve* determined using the *receiver operating characteristic* (AUC-ROC) for the four ML models used.

### 3.3 Hyperparameter tuning and model adaptation

Applying a learning rate of 1.0 × 10^−3^ caused rapid overfitting with very unstable training of the model, while a lower rate of 1.0 × 10^−7^ showed very little training and underfitted (Figure 2). An iterative process of refinement identified 5.0 × 10^−6^ as the optimal rate for model training (Figure 2).

To further improve classification performance, the *ResNet50* architecture was adapted by replacing the final *fully connected* (FC) layer with a custom sequence of dense layers. Each of these layers was combined with batch normalization and the ReLU activation function: (i) layer 1 = linear transformation to 512 features, (ii) batch normalization and ReLU activation (to increase efficiency and stability of the model), (iii) layer 2 = linear transformation to 256 features, (iv) batch normalization and ReLU activation, and (v) layer 3 = final linear transformation to an output neuron for binary classification. This custom extension significantly improved the model’s performance, achieving the highest accuracy (91.57%), precision (0.9286), recall (0.9070), F1 score (0.9176), and AUC-ROC (0.9529) compared to all previously tested models (for code see Supplementary material S1).

### 3.4 Annotated heatmaps and digital health application

**Figure 4:**
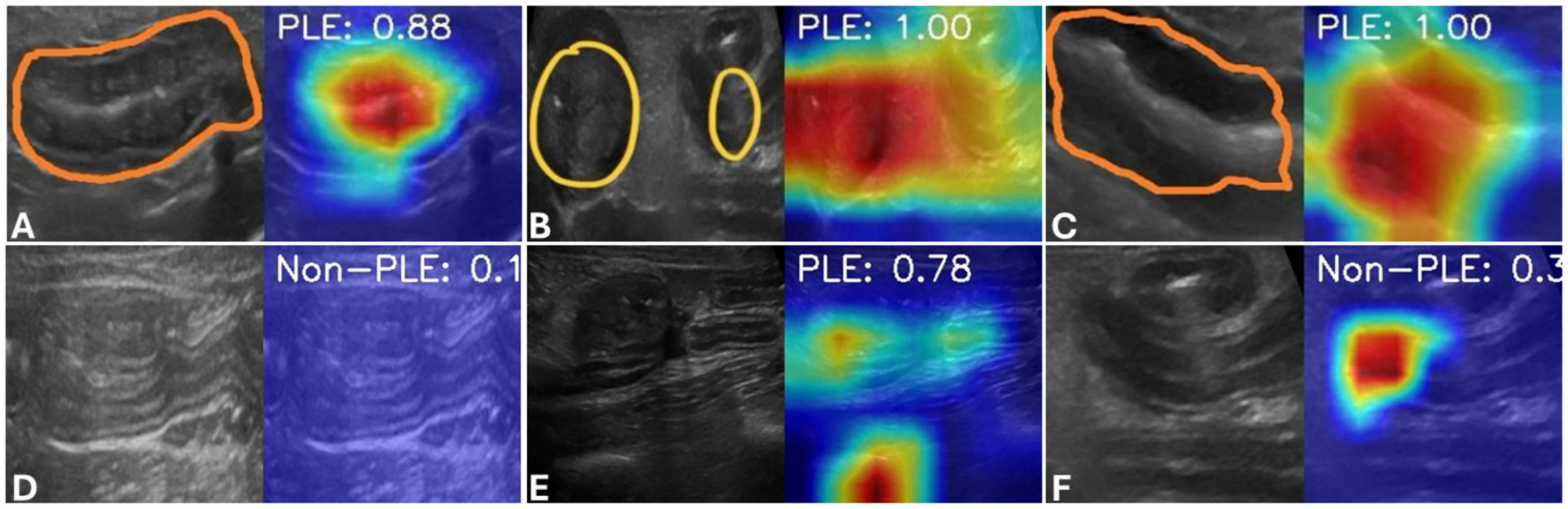
Annotated heatmaps. Sonographic images of select intestinal sections with lesions of PLE **(A–C)** or absence of such lesions in a dog with non-PLE CIE **(D)** are shown. Each image panel includes the sonographic image with manual markings of the relevant areas (ROIs, orange or yellow) on the left (**A**: Increased wall thickness and mucosal striations; **(B)**: increased wall thickness, mucosal hyperechogenicity, ascites; **C**: increased wall thickness and intestinal corrugation) and the corresponding AI-annotated heatmaps with color markings of the relevant areas on the right. Examples are also included for an incorrect classification of non-PLE CIE as PLE **(E)** and of a dog with PLE as non-PLE **(F)**.

Heatmaps showed that the optimized *ResNet50* model was very good at recognizing relevant areas (ROIs), such as intestinal wall changes and accumulations of free fluid (ascites) in the area around the intestine (Figure 4).

The annotations served to verify the accuracy of the model, which could be used in two key clinical applications. As a *diagnostic support*, the model can assess whether the findings support or rule out PLE in dogs and thus function as a diagnostic aid that could be integrated into the current stepwise diagnostic algorithm for CIE and PLE in dogs. The assessment yields a fraction, where 1.00 corresponds to a 100% probability (Figure 4). As a *tool to mark relevant image areas*, the model can highlight suspicious regions containing features of PLE. The digital health application used on a small set of still images obtained from other sources and using other ultrasound machines could correctly classify cases of PLE vs. non-PLE cases with approximately 30% accuracy.

## 4 Discussion

Artificial intelligence (AI) plays an increasingly important role in various areas of everyday life, ranging from applications such as ChatGPT to modern individualized medicine using digital health tools (Rodgers et al., 2023). With the help of digital tools, simple routine tasks and also complex problems may be solved within a very short time. These advantages are increasingly being exploited in medical diagnostics, treatment, and therapy monitoring. However, the use of AI-assisted tools in the medical field also presents challenges and limitations. Particularly in the context of canine PLE, the combination of the clinical relevance of the disease and the lack of standardized diagnostic algorithms makes research into such innovative methods not only technologically exciting, but also urgently necessary in canine clinical medicine. As with any technological innovation, AI cannot work error-free on its own, making human verification by experienced specialists (i.e., validation) essential (Aggarwal et al., 2024; Rohan Krishna et al., 2025). Thus, the combination of AI and professional expertise offers great potential for further optimizing patient care and increasing efficiency in everyday medical practice and clinical settings.

The promising results of this pilot study suggest that the AI model developed could be used as a surrogate tool for the detection of PLE in dogs, either as primary ILE or a more severe form of idiopathic CIE. Early detection of affected canine patients enables early planning of adequate diagnostics and/or therapeutic intervention, thereby helping to optimize the prognosis for an affected dog (Jablonski, 2022; Craven and Washabau, 2019; Caulfield et al., 2021). However, further steps are necessary before such a model can be introduced and recommended for use in clinical practice or veterinary training. These include more comprehensive testing and validation under practical conditions, technical integration into existing diagnostic imaging systems, and the establishment of recommended workflows and diagnostic algorithms. A larger group of experts in diagnostic imaging and veterinarians should be involved to define how the model can be integrated into the diagnostic decision-making process and what significance the results have in diagnostically ambiguous cases.

*ResNet50* belongs to the family of residual networks (He et al., 2016) and is a relatively large and robust model that requires high computing power for training, which extends the training time and requires significant resources (i.e., memory). Because *ResNet50* is a deep network characterized by its ability to recognize complex visual patterns, it appears suitable for integrating complex and potentially artifact-laden sonographic still images (Remedios et al., 2021; Fu et al., 2025). A major advantage of *ResNet* is the introduction of residual connections or skip connections, where not every layer is added to the previous one, but some layers are skipped and added to a deeper layer (Sanchez-Cesteros et al., 2023; Liu et al., 2025). This has the advantage of avoiding information loss and enables filtering highly abstract features from the available data set, allowing such deep networks to learn faster and more efficiently, as only changes need to be actively learned (Ali, 2025).

The potential limitation of the model to slightly overfitting to the specific characteristics of the dataset has to be considered, despite a good performance on the unknown test set (Tetko and Clevert, 2025; Riahi et al., 2025). This means that a model could learn the training data too well, including all confounding factors and irrelevant details (e.g., random patterns such as iatrogenic markers or sections of other abdominal organs that may be present in sonographic still images) (Alnaggar et al., 2024; Galić et al., 2023). An indication for overfitting would be a high training accuracy with significantly poorer validation accuracy (Ali, 2025). Although regularization techniques (e.g., dropout L2 weight decay) were applied, it remains possible that the model learned patterns that are only effective on the same source of ultrasonographic images and might perform different (e.g., worse) on images from different sources. This also requires more study and multicenter validation to more effectively evaluate the capabilities of the model.

Sonographic image data from dogs of various breeds, obtained during routine diagnostic evaluation of dogs at the Internal Medicine service of the Small Animal Clinic at the University of Leipzig, were utilized to develop and validate the model. The analysis included dogs with a suspicion of CIE/PLE that underwent imaging, laboratory, and clinical diagnostics and had complete medical records documentation, as well as ultrasonographic images of sufficient quality. The resulting study population comprised a spectrum of different dog breeds (including mixed-breed dogs), breed sizes (small- to large-breed dogs), and age groups (young adult to older dogs), deliberately chosen to test the robustness of the AI model against breed- and size-related variations. Dogs with incomplete clinical data had to be excluded if a final diagnosis of “PLE” vs. “non-PLE” could not be determined, as the model relies on validated diagnoses. Some dogs could also not be included, despite the availability of imaging data, if the images were of insufficient quality (e.g., the relevant intestinal segment could not be determined, significant artifacts were contained in the images, or a clear interpretation of the images was lacking), as these would have distorted or falsified the ML model training. While the level of attrition in this study increases the risk for selection bias, comparative analysis of the demographics between dogs from which sonographic images were finally included in the analyses (*n* = 59) and dogs from which this was not possible (*n* = 81) did not reveal any differences in terms of age distribution, body weight, sex and neuter status, breed, and proportions of PLE vs. non-PLE dogs, serum albumin and total protein concentrations (all *p* > 0.1).

Still, a significant limitation of this study is the small size of the data set, which could limit the transferability of the results to different devices, users, and veterinary care facilities. Overall, only a limited set of sonographic images from clinical patients with PLE or non-PLE CIE could be included in this pilot study, creating a challenge for patient-level (dog-level) splitting. Small medical datasets are often constrained by the high cost and complexity of data acquisition, and enforcing strict subject-level partitioning can make model training and evaluation impractical (Shaikhina and Khovanova, 2017). This applies to our study, where, given the limited number of samples, applying patient-level splitting would have resulted in insufficient samples per split, severe class imbalance, and unreliable optimization, thereby preventing meaningful model development (Shaikhina and Khovanova, 2017). We therefore adopted image-level splitting as a pragmatic choice for this exploratory study, while explicitly acknowledging our small dataset and the associated risk of correlated images due to random splitting creating the risk of an imbalance in detecting patient- vs. disease-related features and optimistic estimates of accuracy and AUC (Alnaggar et al., 2024; Galić et al., 2023; An et al., 2021). Given these limitations, our results should be viewed as proof-of-concept demonstrations of feasibility, rather than definitive performance metrics.

Expanding the dataset to include a larger number of clinical patients and contributions from different secondary and tertiary veterinary centers would increase the generalizability of the model and possibly further improve its diagnostic accuracy, which appeared limited based on the preliminary external validation of the digital health application. Such a multicenter study—involving various veterinary care facilities and levels, ultrasound machines, operators, and operator experience levels—will provide the context to evaluate whether and how the trained model can be applied to previously unknown datasets (sonographic still images generated during routine diagnostic evaluations) and different pre-test probabilities (levels of suspicion for PLE or non-PLE CIE). From a practical aspect, however, proprietary algorithms incorporating specific ultrasound machines and standardized (single veterinary center, imaging, and operator-based) protocols might still be very attractive and potentially more practical than a perfect general model that can be transferred to any environment. However, this will still require training and validation using large data sets for correct classification.

We acknowledge a further limitation of the study presented by data acquisition and processing. Archived sonographic still images of the intestine were not available for all 157 dogs considered for inclusion in this investigation. In some cases, only a few sections or less adequate (e.g., tangential view, motion artifacts) images of the intestine were available, whereas a number of images could be included for other patients. Because ultrasonography (B-mode) is a cross-sectional imaging technique that uses high-frequency sound waves to visualize anatomical structures, and resolution depends on the frequency of the sound waves, still images are generally inferior to video sequences for retrospective diagnostic evaluation (Parsai et al., 2012; Weimer et al., 2025). Thus, both still images and video sequences could be incorporated into future models, but considerably higher data storage requirements must be considered.

Another limitation is that data were extracted exclusively for the gastrointestinal tract or affected sections of the intestine. Structures such as the kidneys or other abdominal organs were not included in the final training data set, as such irrelevant structures could mislead the model during the training phase and thus affect the results (Matthees et al., 2025). Similarly, inaccurate data (e.g., blurred still images of the intestinal wall, images including measurement markers) were not included in the training of the model. Other factors that impaired training accuracy and led to exclusion of images were the presence of overlapping structures (e.g., ingesta-filled intestine) or artificial distortions (patient-related factors such as motion artifacts or technically unavoidable sonographic artifacts). In addition, PLE features may be focal or multifocally present in the intestine (Jablonski, 2022; Craven and Washabau, 2019). Thus, data from different regions of the intestine can vary in terms of sonographic findings and PLE differentiation. Finally, the training data set of the model included a larger number of non-PLE CIE cases than PLE dogs. This slightly imbalanced population can lead to a biased model if not adequately compensated for during the training of the model (Alnaggar et al., 2024; Galić et al., 2023). Lastly, inter-observer reliability could not be calculated for the annotation of the heatmaps due to employing a consensus evaluation.

Clinical applications of AI-supported image analysis systems have been the subject of intensive research in human medicine for several years (Chen et al., 2025; Decharatanachart et al., 2021). Several studies suggest that deep learning models can achieve equivalent or even superior diagnostic accuracy to human experts when evaluating radiographic, sonographic, or endoscopic images (Lång et al., 2023; Mosch et al., 2022). Thus, AI-supported image analysis lends itself to integration into routine diagnostic algorithms but might also be suitable to detect more complex findings or patterns. However, such approaches are currently understudied in veterinary medicine, particularly for complex conditions such as CIE and PLE. Our pilot study aimed to present a first step toward closing this diagnostic gap.

Integration of an AI-based tool, as evaluated in this study, could be of value in routine diagnostic evaluations by increasing diagnostic certainty and potentially saving time and costs. Ultrasonographic findings in chronic intestinal diseases can be inconsistent and depend on experience, equipment, and image quality. AI-supported identification of disease-relevant patterns at an early stage (e.g., by visually highlighting affected areas using heat maps) has the potential to reduce inter-examiner variability and increase diagnostic consistency. Assessment of patients using an AI-assisted algorithm might also accelerate the diagnostic evaluation in CIE- and/or PLE-suspected dogs and shorten the time to further diagnostic and/or therapeutic decisions, a critical factor as PLE often carries a poor prognosis. Integration of an AI-based tool into diagnostic algorithms could also provide a suitable educational tool and support veterinary students and veterinarians with limited experience in diagnostic ultrasound, particularly in diagnosing canine PLE and/or recognizing and integrating PLE-related features. A possible integration of an explainable AI-based tool in this context could be assistance with decision-making algorithms and AI-based annotation of suspicious intestinal segments via heat maps (e.g., thickened layers of the intestinal wall, altered echogenicity patterns). Thus, the primary target group of such an AI-supported tool would be veterinary clinics with ultrasound equipment that commonly see dogs with gastrointestinal signs, pets and pet owners who might benefit from the incorporation of such tools, and veterinary training centers.

However, integration of an AI-based tool into routine clinical workflows will raise questions concerning the practical implications of false-negative and false-positive outputs and thus liability when arriving at diagnostic, therapeutic, or even prognostic decisions. Thus, further work is also needed to clarify the appropriate clinical context (e.g., prior test probability) for using such AI-based technology in a scientifically adequate and clinically responsible way (i.e., as a diagnostic assistance system rather than a substitutional technology).

## 5 Conclusion

The results of this study demonstrate the potential of integrating an AI-based model into CIE diagnostics and PLE differentiation in canine clinical gastroenterology. By combining sonographic diagnostics with ML, a high level of accuracy can be achieved for PLE differentiation. Future research is needed to expand the database and integrate the model into the currently recommended diagnostic algorithm for CIE-/PLE-suspected dogs. These findings should also stimulate similar research in other areas of image-guided clinical diagnostics, such as endoscopic evaluation.

## Data Availability

The raw data (fully anonymized) supporting the conclusions of this article will be made available by the authors, upon reasonable request.
