# Supplementary material for: Detection of protein-losing enteropathy (PLE) ultrasonographic imaging features in dogs using deep learning neural networks
Source: Front Artif Intell. 2026 Jan 8;8:1707957. doi: 10.3389/frai.2025.1707957 (PMC12823995; doi:10.3389/frai.2025.1707957)
Supplement: Supplementary file 1 [file Data_Sheet_1.PDF]

Supplementary Material

1 Code

In [ ]:
```
####### Installation and Import Setup ####
!pip install torch torchvision torchaudio
!pip install scikit-learn
!pip install torchcam
!pip install grad-cam

#Imports
import torch
import torch.nn as nn
import torch.optim as optim
from torchvision import models, transforms, datasets
from torch.utils.data import DataLoader , WeightedRandomSampler
import matplotlib.pyplot as plt
import os
os.environ["PYTORCH_CUDA_ALLOC_CONF"] = "expandable_segments:True"
```

In [ ]:
```
####### Dataset splitting, Preprocessing and Augmentation ####

import random
import shutil
import os
from pathlib import Path
from PIL import Image, ImageEnhance, ImageOps

def apply_op(image, op_name, magnitude):
    """Applies the requested operation to the image."""
    if op_name == 'rotate':
        return image.rotate(magnitude)

    elif op_name == 'zoom':
        w, h = image.size
        new_w, new_h = int(w * magnitude), int(h * magnitude)
        zoomed = image.resize((new_w, new_h), Image.BICUBIC)

        # If zoomed in, randomly crop back to original size
        if new_w > w and new_h > h:
            max_x = new_w - w
            max_y = new_h - h
            x = random.randint(0, max_x)
            y = random.randint(0, max_y)
            return zoomed.crop((x, y, x + w, y + h))
        return zoomed

    elif op_name == 'brightness':
        enhancer = ImageEnhance.Brightness(image)
        return enhancer.enhance(magnitude)

    return image

def random_augment(image):
    """Randomly applies one of the defined augmentations."""
    operations = {
        'rotate': (0, 360),
        'zoom': (0.8, 1.5),
        'brightness': (0.5, 1.5)
    }
    op_name = random.choice(list(operations.keys()))
    min_val, max_val = operations[op_name]
```

```
    magnitude = random.uniform(min_val, max_val)
    return apply_op(image, op_name, magnitude)

def balance_training_dataset(train_dir):
    """
    Balances the training dataset by duplicating images from the subfolde
    with fewer images until both 'PLE' and 'Non-PLE' have equal counts.
    """
    train_dir = Path(train_dir)
    ple_dir = train_dir / "PLE"
    non_ple_dir = train_dir / "Non-PLE"

    # List all files in the subfolders (assuming each file is an image)
    ple_images = [f for f in ple_dir.iterdir() if f.is_file()]
    non_ple_images = [f for f in non_ple_dir.iterdir() if f.is_file()]

    count_ple = len(ple_images)
    count_non_ple = len(non_ple_images)

    print(f"Current counts -> PLE: {count_ple}, Non-PLE: {count_non_ple}"

    # If counts are equal, nothing to balance
    if count_ple == count_non_ple:
        print("Dataset is already balanced.")
        return

    # Select the folder with fewer images
    if count_ple < count_non_ple:
        lower_dir = ple_dir
        lower_images = ple_images
        target_count = count_non_ple
    else:
        lower_dir = non_ple_dir
        lower_images = non_ple_images
        target_count = count_ple

    difference = target_count - len(lower_images)
    print(f"Balancing dataset by duplicating {difference} images in folde

    # Duplicate random images from the lower-count folder until counts ar
    for i in range(difference):
        chosen_image = random.choice(lower_images)
        # Generate a new filename to avoid overwriting existing files
        new_filename = f"{chosen_image.stem}_dup_{i}{chosen_image.suffix}
        new_path = lower_dir / new_filename
        shutil.copy(chosen_image, new_path)
        lower_images.append(new_path)

    print("Dataset balancing complete.")

def get_dataset_stats(split_dirs, classes):
    """
    Recalculate the dataset statistics by scanning the split directories.
    Returns a new stats dictionary.
    """
    updated_stats = {}
    for cls in classes:
        updated_stats[cls] = {}
        for split, split_dir in split_dirs.items():
            class_dir = split_dir / cls
```

```
            # Get all files in this directory
            files = [f for f in class_dir.iterdir() if f.is_file()]
            # Count original images as those without "_AUG_" or "_BALANCE
            original_count = sum(
                1 for f in files
                if ("_AUG_" not in f.stem and "_BALANCED_" not in f.stem)
            )
            augmented_count = len(files) - original_count
            updated_stats[cls][split] = {"original": original_count, "aug
    return updated_stats

# Set random seed for reproducibility
random.seed(22)

def augment_image(image_path, num_augmentations=7):
    image = Image.open(image_path)
    augmented_images = []
    for _ in range(num_augmentations):
        augmented_images.append(random_augment(image.copy()))
    return augmented_images

# Define paths and parameters
original_root = "PLE_FINAL_DATASET" ## the non split dataset
split_root = "final_dataset"        ## the dataset for training
ifAug = True

# Define dataset splits (train/val/test ratios)
splits = {
    "train": 0.7,
    "val": 0.1,
    "test": 0.2
}

# Ensure original dataset directory exists
if not os.path.exists(original_root):
    raise FileNotFoundError(f"Original dataset directory '{original_root}

# Create split directories if they don't exist
split_dirs = {split: Path(split_root) / split for split in splits.keys()}
for d in split_dirs.values():
    d.mkdir(parents=True, exist_ok=True)

classes = ["PLE", "Non-PLE"]

# Process each class in the original dataset
for cls in classes:
    class_dir = Path(original_root) / cls

    # Verify class directory exists
    if not class_dir.exists():
        raise FileNotFoundError(f"Class directory '{class_dir}' not found

    # Get all valid images
    imgs = [
        f for f in os.listdir(class_dir)
        if os.path.isfile(os.path.join(class_dir, f))
        and f.lower().endswith(('.jpg', '.jpeg', '.png'))
    ]

    if not imgs:
```

```
        raise ValueError(f"No valid images found in {class_dir}")

    random.shuffle(imgs)
    total = len(imgs)

    # Calculate split sizes
    train_count = int(total * splits["train"])
    val_count = int(total * splits["val"])
    test_count = total - train_count - val_count

    # Split images into train, validation, and test sets
    split_imgs = {
        "train": imgs[:train_count],
        "val": imgs[train_count:train_count + val_count],
        "test": imgs[train_count + val_count:]
    }

    # Create class subdirectories in each split folder
    for split_dir in split_dirs.values():
        (split_dir / cls).mkdir(exist_ok=True)

    # Copy images to split directories and augment training images if ena
    for split, images in split_imgs.items():
        for img in images:
            src = class_dir / img
            dst = split_dirs[split] / cls / img
            # Always copy the original image
            shutil.copy2(str(src), str(dst))
            # Perform augmentation for train set if enabled
            if split == "train" and ifAug:
                aug_images = augment_image(src, 10)  # Adjust augmentatio
                for idx, aug_img in enumerate(aug_images):
                    aug_name = f"{Path(img).stem}_AUG_{idx}{Path(img).suf
                    aug_dst = split_dirs[split] / cls / aug_name
                    aug_img.save(aug_dst)

# Balance the training dataset after augmentation
balance_training_dataset(split_dirs["train"])

# Get updated dataset statistics
dataset_stats = get_dataset_stats(split_dirs, classes)

print("\nUpdated dataset split statistics (including original and augment
for cls in classes:
    print(f"\n{cls}:")
    for split, stats in dataset_stats[cls].items():
        print(f"  {split}:")
        print(f"    Original: {stats['original']} images")
        print(f"    Augmented: {stats['augmented']} images")

print(f"\nSplit complete. Datasets (with augmentations in train) saved to
```

```
#######  Data Standard Pipeline #######
from torchvision.transforms import v2

data_transforms = {
    'train': transforms.Compose([
        transforms.Resize((224, 224)),
        transforms.ToTensor(),
        transforms.Normalize(mean=[0.485, 0.456, 0.406], std=[0.229, 0.22
```

```
    ]),
    'val': transforms.Compose([
        transforms.Resize((224, 224)),
        transforms.ToTensor(),
        transforms.Normalize(mean=[0.485, 0.456, 0.406], std=[0.229, 0.22
    ]),
    'test': transforms.Compose([
        transforms.Resize((224, 224)),
        transforms.ToTensor(),
        transforms.Normalize(mean=[0.485, 0.456, 0.406],
                             std=[0.229, 0.224, 0.225])
    ])
}
split_root = "final_dataset"


# Define paths to the split datasets

train_path =  split_root + '/train'
val_path = split_root + '/val'
test_path = split_root + '/test'

# Create datasets using ImageFolder
train_dataset = datasets.ImageFolder(train_path, transform=data_transform
val_dataset = datasets.ImageFolder(val_path, transform=data_transforms['v
test_dataset = datasets.ImageFolder(test_path, transform=data_transforms[

# Calculate class counts for balanced sampling (if needed)
targets = [label for _, label in train_dataset.samples]
class_counts = torch.bincount(torch.tensor(targets))

# Create data loaders
train_loader = DataLoader(train_dataset, batch_size=32, shuffle=True)
val_loader = DataLoader(val_dataset, batch_size=16, shuffle=False)
test_loader = DataLoader(test_dataset, batch_size=16, shuffle = False)

# # Set computation device
device = torch.device("cuda" if torch.cuda.is_available() else "cpu")
print(f"Using device: {device}")

# Print dataset info
print(f"Number of classes: {len(train_dataset.classes)}")
print(f"Class names: {train_dataset.classes}")
print(f"Training samples per class: {dict(zip(train_dataset.classes, clas
```

n [ ]:

```
####### Create Model with Enhanced Architecture #######
from torchvision.models import  ResNet50_Weights, MobileNet_V2_Weights
print("training on: " + split_root)
## Function to create and fine-tune a model (no layers are frozen)
def create_model(arch='mobilenet_v2', pretrained=True, num_classes=1):
    if arch.lower() == 'mobilenet_v2':
        model = models.mobilenet_v2(pretrained = True )
        num_ftrs = model.classifier[1].in_features
        model.classifier = nn.Sequential(
            nn.Dropout(0.5),
            nn.Linear(num_ftrs, num_classes)
        )
    elif arch.lower() == 'resnet50':
        model = models.resnet50(pretrained = True )
        num_ftrs = model.fc.in_features
```

```
        model.fc = nn.Sequential(
            nn.Dropout(0.5),
            nn.Linear(num_ftrs, num_classes)
        )
    elif arch.lower() == 'resnet50custom':
          model = models.resnet50(pretrained = True )
          num_ftrs = model.fc.in_features
          model.fc = nn.Sequential(
              nn.Linear(num_ftrs, 512),
              nn.BatchNorm1d(512),
              nn.ReLU(inplace=True),
              nn.Linear(512, 256),
              nn.BatchNorm1d(256),
              nn.ReLU(inplace=True),
              nn.Linear(256, 1),
          )

    elif arch.lower () == 'alexnet':
        model = models.alexnet(pretrained = True)
        model.classifier = nn.Sequential(
            nn.Dropout(0.5),
            nn.Linear(256 * 6 * 6, 4096),
            nn.ReLU(inplace=True),
            nn.Dropout(0.5),
            nn.Linear(4096, 4096),
            nn.ReLU(inplace=True),
            nn.Linear(4096, 1),
        )

    elif arch.lower () == 'effnet_b0':
         model = models.efficientnet_b0(pretrained = True)
         num_ftrs = model.classifier[1].in_features
         model.classifier = nn.Sequential(   ## for mob and effnet
         nn.Dropout(0.5),
         nn.Linear(num_ftrs, 1)
        )
    else:
        raise ValueError("Unsupported architecture.")
    return model
```

In [ ]:

```
####### Training and Validation Per Epoch Functions #######

def train_one_epoch(model, loader, optimizer, criterion, device):
    model.train()
    running_loss = 0.0
    correct = 0
    total = 0

    for inputs, labels in loader:
        inputs = inputs.to(device)
        labels = labels.float().view(-1, 1).to(device)

        optimizer.zero_grad()
        outputs = model(inputs)


        loss = criterion(outputs, labels)
        loss.backward()

        # # # Gradient clipping to prevent exploding gradients
```

```python
        torch.nn.utils.clip_grad_norm_(model.parameters(), max_norm=0.5)

        optimizer.step()

        running_loss += loss.item()
        predictions = (torch.sigmoid(outputs) > 0.5).float()
        total += labels.size(0)
        correct += (predictions == labels).sum().item()

    epoch_loss = running_loss / len(loader)
    epoch_acc = (correct / total) * 100
    return epoch_loss, epoch_acc

def validate(model, loader, criterion, device):
    model.eval()
    running_loss = 0.0
    correct = 0
    total = 0

    with torch.no_grad():
        for inputs, labels in loader:
            inputs = inputs.to(device)
            labels = labels.float().view(-1, 1).to(device)

            outputs = model(inputs)

            loss = criterion(outputs, labels)
            running_loss += loss.item()

            predictions = (torch.sigmoid(outputs) > 0.5).float()
            total += labels.size(0)
            correct += (predictions == labels).sum().item()

    epoch_loss = running_loss / len(loader)
    epoch_acc = (correct / total) * 100
    return epoch_loss, epoch_acc
```

In [ ]:

```python
####### Training Model with Checkpointing #######

def train_model(split_root, model, train_loader, val_loader, optimizer, c
    best_val_loss = float('inf')
    train_losses, val_losses = [], []
    train_accs, val_accs = [], []
    print("training on: " + split_root)
    model_name = model.__class__.__name__
    print("Model name:", model_name)
    print("Learning rate: " , optimizer.param_groups[0]['lr'])


    for epoch in range(epochs):
         # Train for one epoch and validate afterward
        train_loss, train_acc = train_one_epoch(model, train_loader, opti
        val_loss, val_acc = validate(model, val_loader, criterion, device


        train_losses.append(train_loss)
        val_losses.append(val_loss)
        train_accs.append(train_acc)
        val_accs.append(val_acc)
```

```
        print(f"Epoch {epoch+1}/{epochs}, "
              f"Train Loss: {train_loss:.4f}, Train Acc: {train_acc:.2f}%
              f"Val Loss: {val_loss:.4f}, Val Acc: {val_acc:.2f}%")

         # Save model if validation loss improves
        if val_loss < best_val_loss:
            best_val_loss = val_loss
            print("saved")
            torch.save(model.state_dict(), f'best_{model_name}.pth')

    return train_losses, val_losses, train_accs, val_accs
```

In [ ]:

```
####### Testing and Evaluation of the Model #######
import torch
from torch.utils.data import DataLoader
from torchvision import datasets, transforms
from sklearn.metrics import confusion_matrix, precision_score, recall_sco
import numpy as np

def test_model(model, model_path, test_folder, device, batch_size=16):


    # Load the saved model weights and set to evaluation mode
    model.load_state_dict(torch.load(model_path))
    model.to(device)
    model.eval()

    # Initialize lists to store predictions, probabilities, and true labe
    all_preds = []
    all_probs = []
    all_labels = []

    with torch.no_grad():
        for inputs, labels in test_loader:
            inputs = inputs.to(device)
            labels = labels.to(device)  # For binary classification, ensu

            # Forward pass to get outputs and compute probabilities
            outputs = model(inputs).squeeze(1)
            probs = torch.sigmoid(outputs)  # probabilities in [0,1]
            preds = (probs >= 0.5).long()

            # Append results for later metric computation
            all_preds.extend(preds.cpu().numpy())
            all_probs.extend(probs.cpu().numpy())
            all_labels.extend(labels.cpu().numpy())

    # Compute accuracy and other evaluation metrics
    correct = sum(int(p == l) for p, l in zip(all_preds, all_labels))
    total = len(all_labels)
    test_acc = 100.0 * correct / total

    precision = precision_score(all_labels, all_preds, average='binary',
    recall = recall_score(all_labels, all_preds, average='binary', zero_d
    f1 = f1_score(all_labels, all_preds, average='binary', zero_division=

    cm = confusion_matrix(all_labels, all_preds)

    # Calculate ROC curve and AUC score
    fpr, tpr, thresholds = roc_curve(all_labels, all_probs)
```

```
    roc_auc = auc(fpr, tpr)

    # Print classification report (optional)
    print("Classification Report:")
    print(classification_report(all_labels, all_preds, zero_division=0))

    print(f"Test Accuracy:  {test_acc:.2f}%")
    print(f"Precision:      {precision:.4f}")
    print(f"Recall:         {recall:.4f}")
    print(f"F1-Score:       {f1:.4f}")
    print(f"AUC:            {roc_auc:.4f}")
    print("Confusion Matrix:\n", cm)

    return {
        "test_accuracy": test_acc,
        "precision": precision,
        "recall": recall,
        "f1_score": f1,
        "auc": roc_auc,
        "confusion_matrix": cm.tolist(),
        "fpr": fpr,
        "tpr": tpr,
        "thresholds": thresholds,
        "all_labels": np.array(all_labels),
        "all_probs": np.array(all_probs)
    }
```

In [ ]:

```
####### Train and Test Models #######

def train_and_test_model(split_root, model, device, epochs):

    model_name = model.__class__.__name__
    criterion = nn.BCEWithLogitsLoss()
    optimizer = optim.Adam(model.parameters(), lr=0.000005 , weight_decay

    # Train the model using the previously defined train_model function
    train_losses, val_losses, train_accs, val_accs = train_model(split_ro

    print("Training complete. Now testing the best saved model.")
    test_metrics = test_model(model, f'best_{model_name}.pth', test_path,
    return train_losses, val_losses, train_accs, val_accs, test_metrics



####################
# Train all models##
###################

split_root = "final_dataset"
epochs = 100
device = "cuda"



# First model: ResNet50
torch.cuda.empty_cache()
model = create_model('resnet50')
model = model.to(device)
```

```
train_losses, val_losses, train_accs, val_accs, test_metrics = train_and_
print(test_metrics)

# Free memory after first model
del model
import gc
gc.collect()
torch.cuda.empty_cache()

# Second model: AlexNet
model = create_model('alexnet')
model = model.to(device)
train_losses_alex, val_losses_alex, train_accs_alex, val_accs_alex, test_
print(test_metrics_alex)

# Free memory after second model
del model
gc.collect()
torch.cuda.empty_cache()

# Third model: Effnet_b7
model = create_model('effnet_b0')
model = model.to(device)
train_losses_effnet, val_losses_effnet, train_accs_effnet, val_accs_effne
print(test_metrics_effnet)

# Free memory after third model
del model
gc.collect()
torch.cuda.empty_cache()

# Fourth model: MobileNet_v2
model = create_model('mobilenet_v2')
model = model.to(device)
train_losses_mobnet, val_losses_mobnet, train_accs_mobnet, val_accs_mobne
print(test_metrics_mobnet)

# Final cleanup
del model
gc.collect()
torch.cuda.empty_cache()


# Uncomment the following block to train a custom model based on ResNet50
# model = create_model('resnet50custom')
# model = model.to(device)
# train_losses_custom, val_losses_custom, train_accs_custom, val_accs_cus
# print(test_metrics_custom)
#
# Final cleanup for custom model
# del model
# gc.collect()
# torch.cuda.empty_cache(
```

In [ ]:

```
####K-fold Cross-validation (not really used)
from sklearn.model_selection import KFold
```

```python
def run_k_fold(k=5):
    dataset_size = len(train_dataset)
    indices = list(range(dataset_size))
    kfold = KFold(n_splits=k, shuffle=True, random_state=42)

    fold_results = []
    for fold, (train_ids, val_ids) in enumerate(kfold.split(indices)):
        print(f"Starting fold {fold+1}")

        # Create fold-specific samplers
        train_subsampler = torch.utils.data.SubsetRandomSampler(train_ids
        val_subsampler = torch.utils.data.SubsetRandomSampler(val_ids)

        train_loader = DataLoader(train_dataset, batch_size=32, sampler=t
        val_loader = DataLoader(train_dataset, batch_size=16, sampler=val

        model = create_model().to(device)
        optimizer = optim.Adam(model.parameters(), lr=0.000005 , weight_d


        # Train for this fold
        best_fold_val_loss = float('inf')
        for epoch in range(50):  # Reduced epochs for k-fold
            train_loss, train_acc = train_one_epoch(model, train_loader,
            val_loss, val_acc = validate(model, val_loader, criterion, de

            if val_loss < best_fold_val_loss:
                best_fold_val_loss = val_loss
                best_fold_acc = val_acc

        fold_results.append(best_fold_acc)
        print(f"Fold {fold+1} best validation accuracy: {best_fold_acc:.2

    mean_acc = np.mean(fold_results)
    std_acc = np.std(fold_results)
    print(f"\nK-fold cross-validation results:")
    print(f"Mean accuracy: {mean_acc:.2f}% ± {std_acc:.2f}%")
    return mean_acc, std_acc

mean_acc, std_acc = run_k_fold(k=5)
```

n [ ]:

```python
####### Display Model Metrics #######
import matplotlib.pyplot as plt
import matplotlib.gridspec as gridspec
import seaborn as sns
import pandas as pd

def display_model_metrics(model_name, train_losses, val_losses, train_acc

    # Create a figure with a GridSpec layout
    plt.figure(figsize=(12, 10))
    gs = gridspec.GridSpec(2, 2, height_ratios=[1, 1.2])

    # Loss vs. Epoch subplot
    ax0 = plt.subplot(gs[0, 0])
    ax0.plot(train_losses, label='Train Loss')
    ax0.plot(val_losses, label='Val Loss')
    ax0.set_xlabel('Epoch')
    ax0.set_ylabel('Loss')
    ax0.legend()
```

```
    ax0.set_title(f"{model_name} - Loss vs. Epoch")

    # Accuracy vs. Epoch subplot
    ax1 = plt.subplot(gs[0, 1])
    ax1.plot([acc / 100 for acc in train_accs], label='Train Acc')
    ax1.plot([acc / 100 for acc in val_accs], label='Val Acc')
    ax1.set_xlabel('Epoch')
    ax1.set_ylabel('Accuracy')
    ax1.set_ylim(0, 1)
    ax1.legend()
    ax1.set_title(f"{model_name} - Accuracy vs. Epoch")

    # ROC Curve subplot spanning the bottom row
    ax2 = plt.subplot(gs[1, :])
    ax2.plot(metrics['fpr'], metrics['tpr'], color='darkorange', lw=2,
             label=f'ROC curve (AUC = {metrics["auc"]:.4f})')
    ax2.plot([0, 1], [0, 1], color='navy', lw=2, linestyle='--')
    ax2.set_xlabel('False Positive Rate')
    ax2.set_ylabel('True Positive Rate')
    ax2.set_title(f"{model_name} - ROC Curve")
    ax2.legend(loc="lower right")

    plt.tight_layout()
    plt.show()

    # Confusion Matrix
    plt.figure(figsize=(6, 5))
    cm = metrics['confusion_matrix']
    sns.heatmap(cm, annot=True, fmt='d', cmap='Blues',
                xticklabels=test_dataset.classes,
                yticklabels=test_dataset.classes)
    plt.xlabel('Predicted Label')
    plt.ylabel('True Label')
    plt.title(f"{model_name} - Confusion Matrix")
    plt.show()

    # Display summary metrics in a table
    metrics_dict = {
        "Test Accuracy (%)": [metrics["test_accuracy"]],
        "Precision": [metrics["precision"]],
        "Recall": [metrics["recall"]],
        "F1-Score": [metrics["f1_score"]],
        "AUC": [metrics["auc"]]
    }
    metrics_table = pd.DataFrame(metrics_dict)
    print(f"Summary Metrics for {model_name}:")
    print(metrics_table)

models_metrics = {
    "ResNet50": {
        "train_losses": train_losses,
        "val_losses": val_losses,
        "train_accs": train_accs,
        "val_accs": val_accs,
        "metrics": test_metrics
    },
    "AlexNet": {
        "train_losses": train_losses_alex,
        "val_losses": val_losses_alex,
        "train_accs": train_accs_alex,
```

```
        "val_accs": val_accs_alex,
        "metrics": test_metrics_alex
    },
    "EfficientNet_B0": {
        "train_losses": train_losses_effnet,
        "val_losses": val_losses_effnet,
        "train_accs": train_accs_effnet,
        "val_accs": val_accs_effnet,
        "metrics": test_metrics_effnet
    },
    "MobileNet_V2": {
        "train_losses": train_losses_mobnet,
        "val_losses": val_losses_mobnet,
        "train_accs": train_accs_mobnet,
        "val_accs": val_accs_mobnet,
        "metrics": test_metrics_mobnet
    }
}

# Loop through each model and display its metrics
for model_name, data in models_metrics.items():
    display_model_metrics(model_name,
                          data["train_losses"],
                          data["val_losses"],
                          data["train_accs"],
                          data["val_accs"],
                          data["metrics"],
                          test_dataset)
```

In [ ]:

```
import os
import cv2
import torch
import numpy as np
import torch.nn as nn
import torchvision.models as models
from PIL import Image
import torchvision.transforms as transforms

from pytorch_grad_cam import GradCAM
from pytorch_grad_cam.utils.model_targets import ClassifierOutputTarget
from pytorch_grad_cam.utils.image import show_cam_on_image
#####################################
### Grad-CAM for resnet50 ###########
#####################################



# ----------------------------------------------------------------------
# 1) Define Model Architecture Exactly as Trained or as in the weights
# ----------------------------------------------------------------------
model = models.resnet50(pretrained=False)
num_ftrs = model.fc.in_features

model.fc = nn.Sequential(
    nn.Dropout(0.5),
    nn.Linear(num_ftrs, 1)
)

# 2) Load weights
checkpoint_path = "best_ResNet.pth"
```

```
state_dict = torch.load(checkpoint_path, map_location="cpu")

model.load_state_dict(state_dict)
model.eval()

# (Optionally move to GPU)
device = torch.device("cuda" if torch.cuda.is_available() else "cpu")
model.to(device)

# --------------------------------------------------
# 3) Define the Grad-CAM layer (the last conv layer)
# --------------------------------------------------
target_layers = [model.layer4[-1].conv3]
cam = GradCAM(model=model, target_layers=target_layers)

# ------------------------------------------------
# 4) Transforms for input images (typical ResNet)
# ------------------------------------------------
transform = transforms.Compose([
    transforms.Resize((224, 224)),
    transforms.ToTensor(),
    transforms.Normalize(
        mean=[0.485, 0.456, 0.406],
        std =[0.229, 0.224, 0.225]
    )
])

# ----------------------------------------------------
# 5) Loop through test images (including subfolders),
#    predict, generate Grad-CAM, and save side-by-side
# ----------------------------------------------------
test_folder   = split_root + "/test"  # folder with subfolders (PLE, Non_
output_folder = "gradcams"      # folder to save Grad-CAM results
os.makedirs(output_folder, exist_ok=True)

for root, dirs, files in os.walk(test_folder):
    for filename in files:
        image_path = os.path.join(root, filename)
        raw_img = Image.open(image_path).convert("RGB")

        # Preprocess
        input_tensor = transform(raw_img).unsqueeze(0).to(device)

        # Forward pass: output shape [1, 1] for single logit
        output = model(input_tensor)
        prob_ple = torch.sigmoid(output)[0].item()
        is_ple   = (prob_ple >= 0.5)
        pred_label = "PLE" if is_ple else "Non-PLE"

        # -------------------------------
        # Generate Grad-CAM for logit 0
        # -------------------------------
        targets = [ClassifierOutputTarget(0)]
        grayscale_cam = cam(input_tensor=input_tensor, targets=targets)[0

        # ---------------------------------------------
        # Create the overlay visualization (Grad-CAM)
        # ---------------------------------------------
        resized_img = raw_img.resize((224, 224))          # match 224x224
        rgb_img = np.array(resized_img) / 255.0           # float [0..1]
```

```
visualization = show_cam_on_image(
    rgb_img,
    grayscale_cam,
    use_rgb=True
)  # returns an RGB image in uint8 [0..255]

# -----------------------------------
# Convert both images to OpenCV BGR
# -----------------------------------
# 1) The original image in BGR
#    convert from [0..1] float or from PIL.
original_bgr = cv2.cvtColor(np.array(resized_img), cv2.COLOR_RGB2

# 2) The Grad-CAM overlay in BGR
visualization_bgr = cv2.cvtColor(visualization, cv2.COLOR_RGB2BGR

# -----------------------------------
# Add prediction text to the overlay
# -----------------------------------
text = f"{pred_label}: {prob_ple:.2f}"
cv2.putText(
    visualization_bgr,
    text,
    (10, 30),
    cv2.FONT_HERSHEY_SIMPLEX,
    1.0,
    (255, 255, 255),
    2
)

# ----------------------------------------------------
# Concatenate the ORIGINAL and OVERLAY images side by side
# ----------------------------------------------------
side_by_side = cv2.hconcat([original_bgr, visualization_bgr])

# ------------------------------------------------
# Mirror the subfolder structure in output_folder
# ------------------------------------------------
relative_subdir = os.path.relpath(root, start=test_folder)
output_subdir = os.path.join(output_folder, relative_subdir)
os.makedirs(output_subdir, exist_ok=True)

# Build output filename
base_name = os.path.splitext(filename)[0]
out_name  = f"{base_name}_{pred_label}_{prob_ple:.2f}.jpg"
out_path  = os.path.join(output_subdir, out_name)

# Save the final combined image
cv2.imwrite(out_path, side_by_side)

print(f"Processed {image_path} -> {pred_label}, prob={prob_ple:.2
```
